# Exploring the relationships between physiochemical properties of nanoparticles and cell damage to combat cancer growth using simple periodic table-based descriptors

**DOI:** 10.3762/bjnano.15.27

**Published:** 2024-03-12

**Authors:** Joyita Roy, Kunal Roy

**Affiliations:** 1 Drug Theoretics and Cheminformatics Laboratory, Department of Pharmaceutical Technology, Jadavpur University, Kolkata, 700032, India

**Keywords:** cancer cell treatment, cell damage, MeOx NMs (metal oxide nanomaterials), nano-QSPR, zeta potential

## Abstract

A comprehensive knowledge of the physical and chemical properties of nanomaterials (NMs) is necessary to design them effectively for regulated use. Although NMs are utilized in therapeutics, their cytotoxicity has attracted great attention. Nanoscale quantitative structure–property relationship (nano-QSPR) models can help in understanding the relationship between NMs and the biological environment and provide new ways for modeling the structural properties and bio-toxic effects of NMs. The goal of the study is to construct fully validated property-based models to extract relevant features for estimating and influencing the zeta potential and obtaining the toxicity profile regarding cell damage in the treatment of cancer cells. To achieve this, QSPR modeling was first performed with 18 metal oxide (MeOx) NMs to measure their materials properties using periodic table-based descriptors. The features obtained were later applied for zeta potential calculation (imputation for sparse data) for MeOx NMs that lack such information. To further clarify the influence of the zeta potential on cell damage, a QSPR model was developed with 132 MeOx NMs to understand the possible mechanisms of cell damage. The results showed that zeta potential, along with seven other descriptors, had the potential to influence oxidative damage through free radical accumulation, which could lead to changes in the survival rate of cancerous cells. The developed QSPR and quantitative structure–activity relationship models also give hints regarding safer design and toxicity assessment of MeOx NMs.

## Introduction

Engineered nanoparticles have become an integral part of our daily lives in consumable products and commercial goods. Their versatile tunable properties have made nanomaterials a center of innovation in different areas [1]. However, the innovation of nanomaterials (NMs) is hindered because of potential adverse effects. It is believed that small particles can enter the body through inhalation, ingestion, and skin penetration and have the potency to interact with macromolecules for a long period. Many studies have demonstrated that metal oxide nanoparticles (MeOx NPs) are toxic and tend to have adverse effects on living organisms and the environment [2–6]. The toxicity of NPs depends on various structural (intrinsic) [7] and extrinsic properties. Depending on the dispersing environment, nanoparticles can easily agglomerate into particles with larger diameter. Upon intake by organisms, depending on the pH value, these agglomerations disintegrate again becoming a source for toxins in the body [8]. The formation of agglomerated NPs depends upon the surface charge of the NPs, which is believed to stabilize and prevent agglomeration of NPs. As no experimental techniques are available to measure the surface charge directly, its value is measured through the zeta potential (ζ) in a given medium [9]. Zeta potential is the electrostatic potential at the electrical double layer surrounding the NPs in solution. It is closely related to suspension stability and morphology. In metals, the zeta potential can be altered by altering pH, concentration, and conductivity of the components of NPs [10]. Zeta potential can provide information regarding the fate, behavior, and toxicity of NPs in the environment as well as in biological systems. Since the cell membrane is negatively charged, the interaction between NPs and cell membrane or organelles can be highly influenced by the zeta potential. There is an increased interest in integrating data on metal oxides in the field of nanotoxicology that would be able to predict toxicity based on measured properties. Indeed, there are several studies related to the zeta potential and its behavior in solutions and biological systems [11]. Comparable zeta potential measurements across various studies may allow one to find correlations regarding the behavior of different types of NMs. These correlations can then enable the prediction of the behavior of novel NMs based on their properties. As the zeta potential is a system-dependent extrinsic property, it depends on both particle and medium. The behavior of NPs can also change depending on the formation of a protein corona. The formation of a protein corona on the surface of NPs, which influences the interaction with cell membranes or proteins, is also associated with zeta potential and surface charge. Very limited studies have reported the influence of zeta potential, surface charge, hydrophobicity, and biocompatibility on NP toxicity. These properties of NPs determine their toxicity and interaction with the cell membrane damaging human health and the environment [12]. The toxic effect of NPs can be used as a medical treatment for diseases at the cellular level, that is, targeting and destroying cancerous cells. To date, few studies have reported on the mechanism of apoptosis of cancerous cells after metal oxide treatment, which still remains unclear. Traditional approaches are very costly, time-consuming, involve a lot of resources and lead to ethical implications; also, they are inadequate in addressing the safety concerns regarding new NPs in this rapidly growing field. Therefore, computational-based approaches are effective methods in risk assessment. Among them, quantitative structure–property relationship (QSPR) models seem to be the most promising method [13]. However, the physicochemical and structural diversity of metal oxide nanoparticles (MeOx NPs) poses significant challenges in determining their toxic effect on living cells [14–15]. Works related to nanoscale toxicity modeling have been published [16–20] to predict the toxicity profile of MeOx NPs on various cell lines and species. The most important criterion to improve nanoscale toxicity models is the selection of the appropriate structural descriptors of NPs. Periodic table-based descriptors have been a promising tool in predicting toxicity profiles and risk assessment of MeOx NPs with high predictivity and interpretability [21–25]. This type of descriptors can indicate relevant features and mend the mechanism interpretation. Some properties (size, zeta potential, molecular weight, mass percentage of metal elements, and cation charge) are investigated to have a better understanding of the structure of NPs and its influence on toxicity.

## Methods and Materials

### Dataset

The study is based on two datasets, that is, dataset I (zeta potential) and dataset II (cell membrane damage). Dataset I consists of 18 metal oxide nanoparticles (MeOx NPs) with stoichiometries of MO, MO_2_, MO_3_, M_2_O_3_, and M_3_O_4_. This data was obtained from Cao et al. [26], where the zeta potential of MeOx NPs was measured in a cell culture of 20% fetal bovine complete medium. Dataset II was taken from Toropova et al. [27], where cell damage measurement was performed based on the uptake of propidium iodide (PI). The dataset is related to four doses (50, 100, 150, and 200 μg/mL) and exposure times ranging from 1 to 7 h, which results in 132 MeOx NPs data points. The detailed dataset is provided in Supporting Information File 1, Section S1.

### Descriptor calculation

Selecting the appropriate descriptors is crucial for property and toxicity modeling. Quantitative values of chemical features (descriptors) play a significant role in determining the target endpoint. Therefore, in this study, we have calculated periodic table-based descriptors (PT descriptors) for calculating the relevant features contributing to the respective property and toxicity endpoint. Physicochemical features encoding the information of MeOx NPs into PT descriptors were used to build prediction models for zeta potential and cytotoxicity (cell damage). The basic information of MeOx NPs was directly taken from the periodic table and some were calculated with the Elemental Descriptor Calculator software available from (https://sites.google.com/jadavpuruniversity.in/dtc-lab-software/other-dtc-lab-tools?authuser=0), termed first-generation periodic table descriptors. Also, second-generation PT descriptors were calculated using relevant formulas [28]. These descriptors were calculated without any expert intervention and are independent of size variations.

### Splitting of the data sets

Splitting of the datasets into training sets and test sets is essential for developing statistically robust nano-QSPR models. Each of the datasets, that is, the zeta potential dataset and cell damage dataset, was divided into training and test sets with a ratio of 7:3 using the dataset division software in the DTC lab software suite (http://teqip.jdvu.ac.in/QSAR_Tools/). Accordingly, thirteen compounds were in the training set and five compounds in the test set for the zeta potential dataset; for the cell damage dataset, 111 compounds were present in the training set, and the remaining 21 compounds were in the test set. The training set compounds were used for feature selection and model development; the test set was utilized for assessing the predictivity of the developed model.

### Model development

#### Zeta potential QSPR model

To develop the property-based QSPR model, the training set was utilized for model development. The training set of 13 compounds was processed through feature selection via stepwise regression and genetic algorithm (GA) [29]. After feature selection, the training set was utilized for model development through stepwise regression using the MINITAB software (Minitab Inc., USA, https://www.minitab.com). A multiple linear regression (MLR) model was obtained with three descriptors keeping the *F* values to enter and remove 4 and 3.9, respectively. Finally, a PLS (partial least squares) model was developed with the selected features from the MLR model. The developed PLS model consisted of 1 LV (latent variable), which was also developed in the MINITAB software.

#### Cell damage QSPR model

The previously developed QSPR model (dataset I) was utilized to calculate the zeta potential of the MeOx NPs in the cell damage dataset (dataset II), which lacks the zeta potential information (imputation of sparse data). The zeta potential was used as a descriptor in the model development along with the PT descriptors. Although the solvents used for testing metal oxides in both datasets differ, the work involves correlating the zeta potential data (experimental or computed) with the cell damage model as a descriptor. Cao et al. [26] also used zeta potential as one of the determinants for the modeled endpoint. The zeta potential of all data points was determined in the same solvent, and this does not contribute to the variations in zeta potential values due to solvents. This work is similar to imputation in quantitative structure–activity relationship (QSAR) modeling, where a missing value is replaced by a predicted value from another model [30]. The training set with 111 MeOx NPs after feature selection through GA was further used for model development. The model development was performed with stepwise regression using the MINITAB software followed by the best subset selection method. Further, to enhance the quality of predictions for the test set, we have performed a chemical read-across approach for the developed MLR model with eight descriptors.

### Model validation

The validation procedure is the prerequisite for the application of nano-QSPR models. Rigorous validation of the developed models was performed following principles of the Organization for Economic Cooperation and Development [31]. Validation of the model includes both internal and external validation. Internal validation indicates the robustness and fit of the developed model applying the training set, whereas the test set indicates the predictivity of the developed model for new NMs. Common internal validation methods include the leave-one-out cross-validation (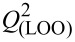) algorithm and the Y-randomization test [32–33]. The model fit ability is expressed by the determination coefficient (*R*^2^) and mean absolute error (MAE). For judging the external predictivity for the test set, we chose the 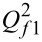 and 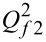 metrics. According to Golbraikh and Tropsha [34], *R*^2^ should be greater than 0.6 and 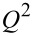 should be greater than 0.5 to meet the standard requirements of external validation. A true external set was also used to evaluate the predicting power of the model. This was done using the prediction reliability indicator (PRI) tool available from the DTC lab software tools (http://teqip.jdvu.ac.in/QSAR_Tools/). To further validate model 2 for the similarity-based prediction, we have performed chemical read-across analysis.

#### Prediction reliability indicator (PRI) tool

**Figure 1:**
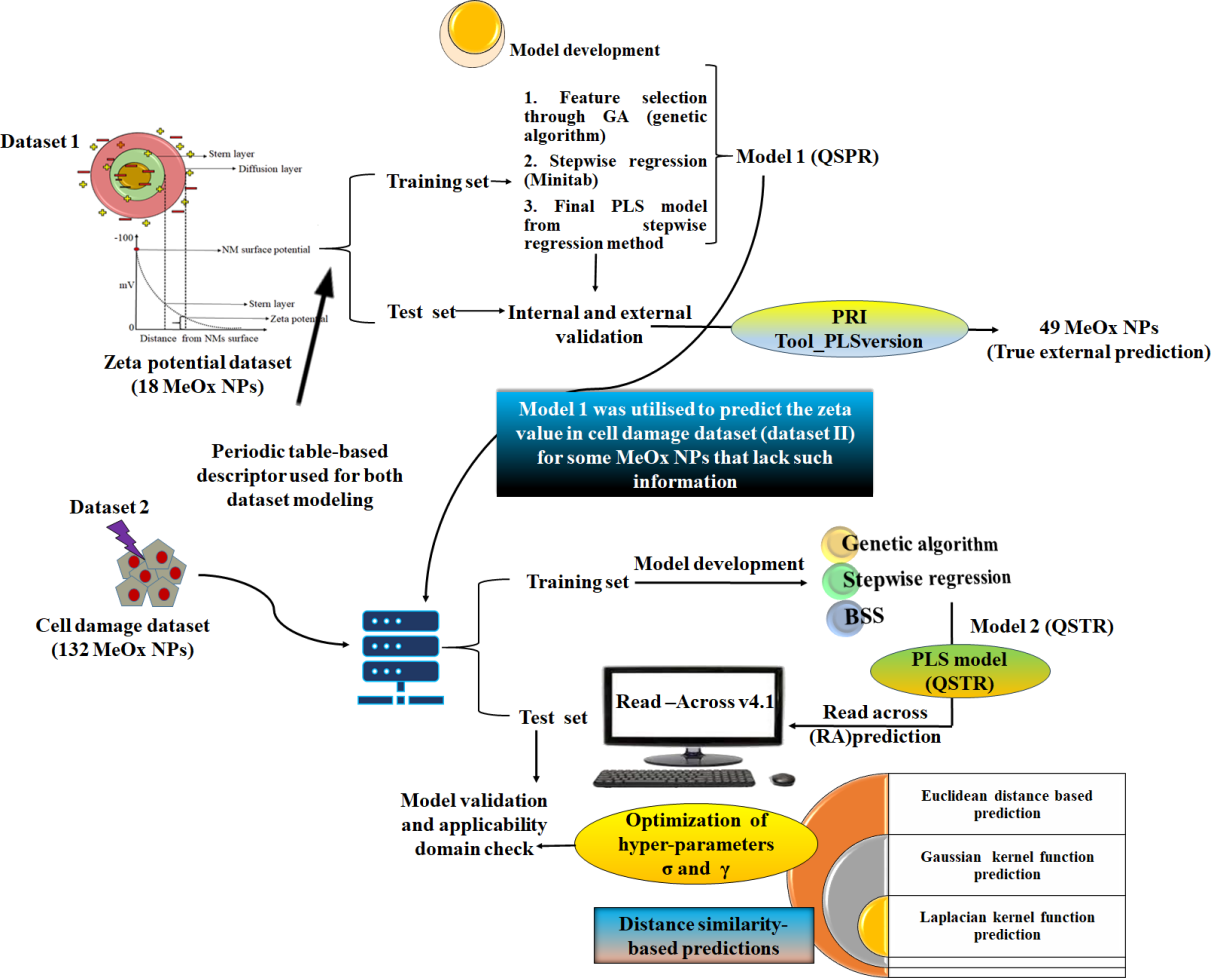
Workflow for developing QSPR (model 1) and QSAR (model 2) models.

Ensuring the reliability of predictions for a new set of data is a vital task. By making robust predictions based on molecular features, we can estimate the external set accurately. In this study, we used the Prediction Reliability Indicator tool [35] (http://teqip.jdvu.ac.in/QSAR_Tools/) to predict the response of a true external set comprising 49 MeOx NPs. The tool categorizes the prediction quality as good, moderate, or bad, based on certain scoring rules. To assess the predictive power of the developed QSPR models, we used the QSPR model (model 1 with zeta potential endpoint) to predict the response of the external set. Figure 1 shows the overall workflow of the present work, highlighting our confident approach to the study.

#### Read-across analysis

The read-across technique is a reliable and scientifically proven method to predict the endpoint of a new compound, also known as the target compound. This technique involves utilizing data from similar substances that have a regular pattern resulting in structural similarity and similar physicochemical, toxicokinetic, toxicodynamic, and ecotoxicological properties [36]. Therefore, after selecting the appropriate descriptors from the PLS model (model 2), we have applied the Quantitative Read-Across v4.0 tool available from our laboratory website (https://sites.google.com/jadavpuruniversity.in/dtc-lab-software/home). This tool uses a similarity-based approach based on Euclidean distance, Gaussian kernel function, and Laplacian kernel function. The method requires optimization of the hyperparameters (sigma and gamma values, distance, and similarity thresholds). To ensure the best results, we used dataset 2, which we divided into a 70% training set and a 30% test set. We further divided the training set into a sub-training and sub-test set to fine-tune the hyperparameters by changing the default setting. Finally, we used the best hyperparameters to predict the external set and achieved the best possible results through a rigorous process.

### Applicability domain

A nano-QSPR model should have a clear range of applicability domains [37]. Robustness and predictivity regarding new compounds are based on the similar physicochemical properties of the compounds in the training set, depending on which, the model chemical space is developed. In the present study, the commonly used Williams plot [38] method was employed to determine whether the compound is within the chemical domain of the model or outside. The vertical axis represents cross-validated standardized residuals whereas the horizontal axis represents leverage values (*h*). This index measures the similarity between the new chemicals and the ones in the training set. The compound prediction is said to be reliable if *h* is less than the critical value (h*). Here, h* is the warning leverage in the Williams plot or applicability domain; compounds lying above this critical value are considered as outliers. The critical leverage h* is calculated as h* = 3*p*/*n*, where *p* stands for the number of modeled variables plus one and *n* stands for the data size of the training set used in model development. Compounds with a cross-validation standardized residual greater than three standard deviations can be considered as Y-outliers.

## Results and Discussion

**Figure 2:**
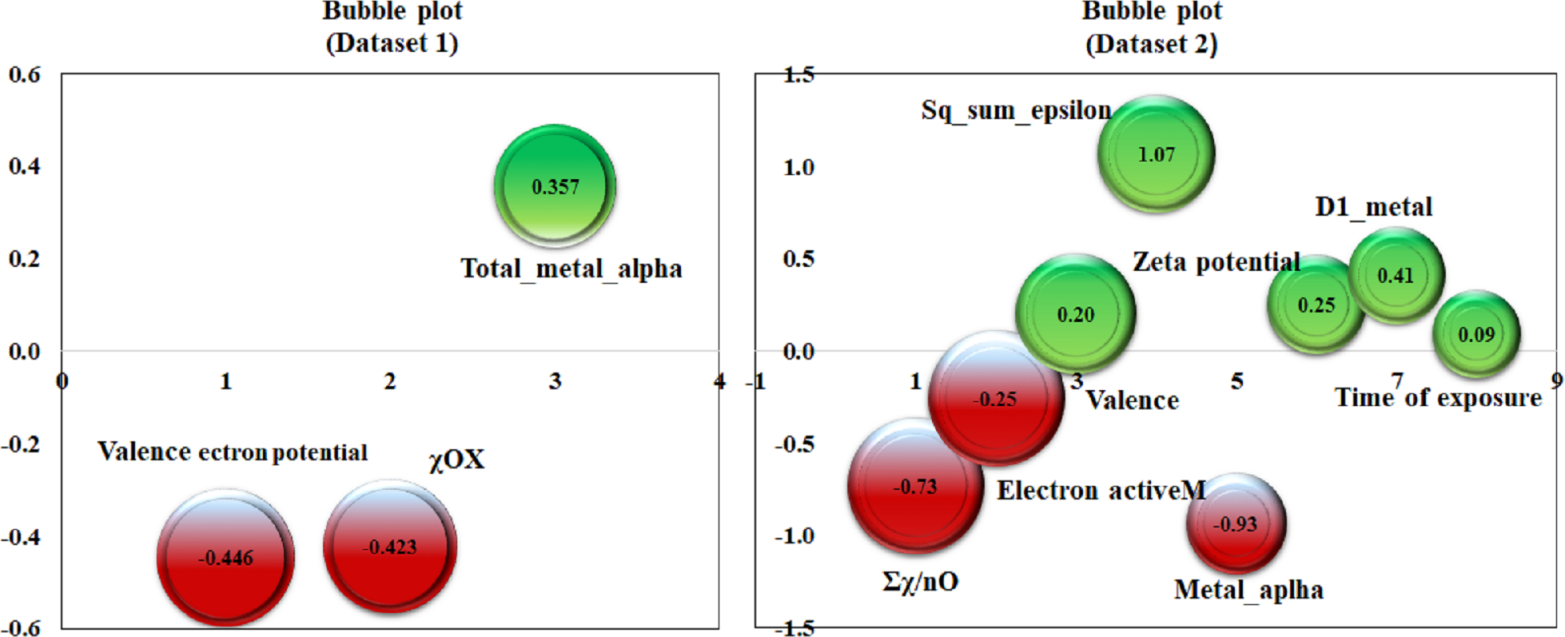
Bubble plot for dataset 1 (model 1) and dataset 2 (model 2).

**Figure 3:**
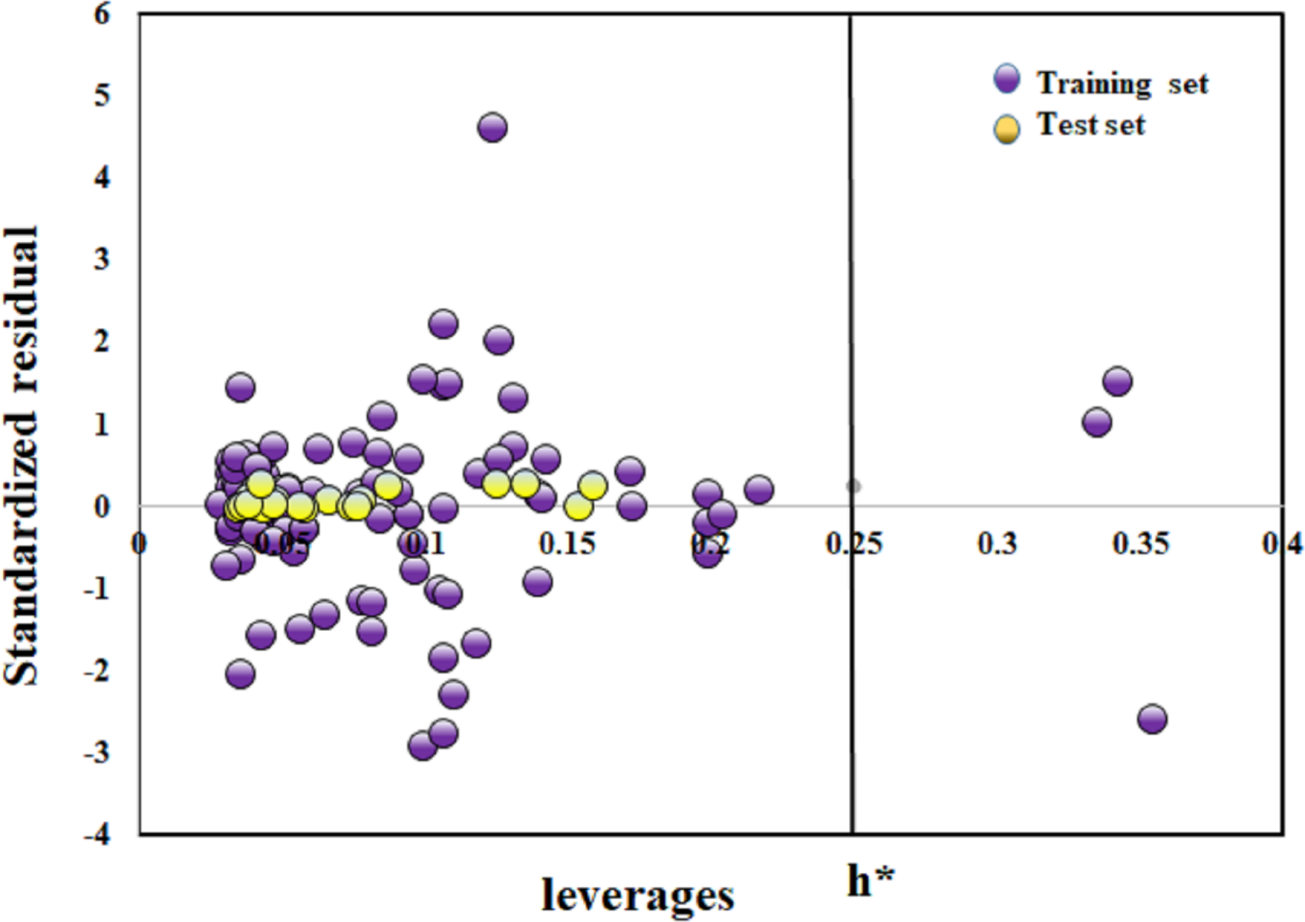
Williams plot for cell damage endpoint (model 2).

To explore the physiochemical properties influencing the zeta potential of the MeOx NPs, property-based modeling was performed considering the zeta potential as the Y-response (model 1). Model 1 was developed with basic periodic table-based descriptors. The different validation metrics showed the models to be robust and of good predictivity. Furthermore, toxicity-based modeling (model 2) was conducted to illustrate the impact of zeta potential on BEAS-2B cell damage. The modeling aimed to create robust and predictive property- and toxicity-based models capable of predicting novel MeOx NPs with enhanced features. Figure 2 shows the bubble plots for both dataset 1 and dataset 2. The green and red colors indicate the positive and negative coefficients of the respective descriptors. The size of the bubble represents the importance of the descriptors; smaller bubbles indicate less contribution to the respective endpoints than larger bubbles. The Y-randomization plot and loading plot are also reported in Supporting Information File 2 and Supporting Information File 1, Figure S1 and Figure S2. The Williams plot in Figure 3 shows that three compounds were outliers in the cell damage dataset. According to the PRI tool estimation on a true external set, out of 49 MeOx NPs, we confidently predicted 39 with good accuracy using this simple tool. This means that we were able to make predictions for untested metal oxides with great confidence.

### QSPR model for zeta potential

**Figure 4:**
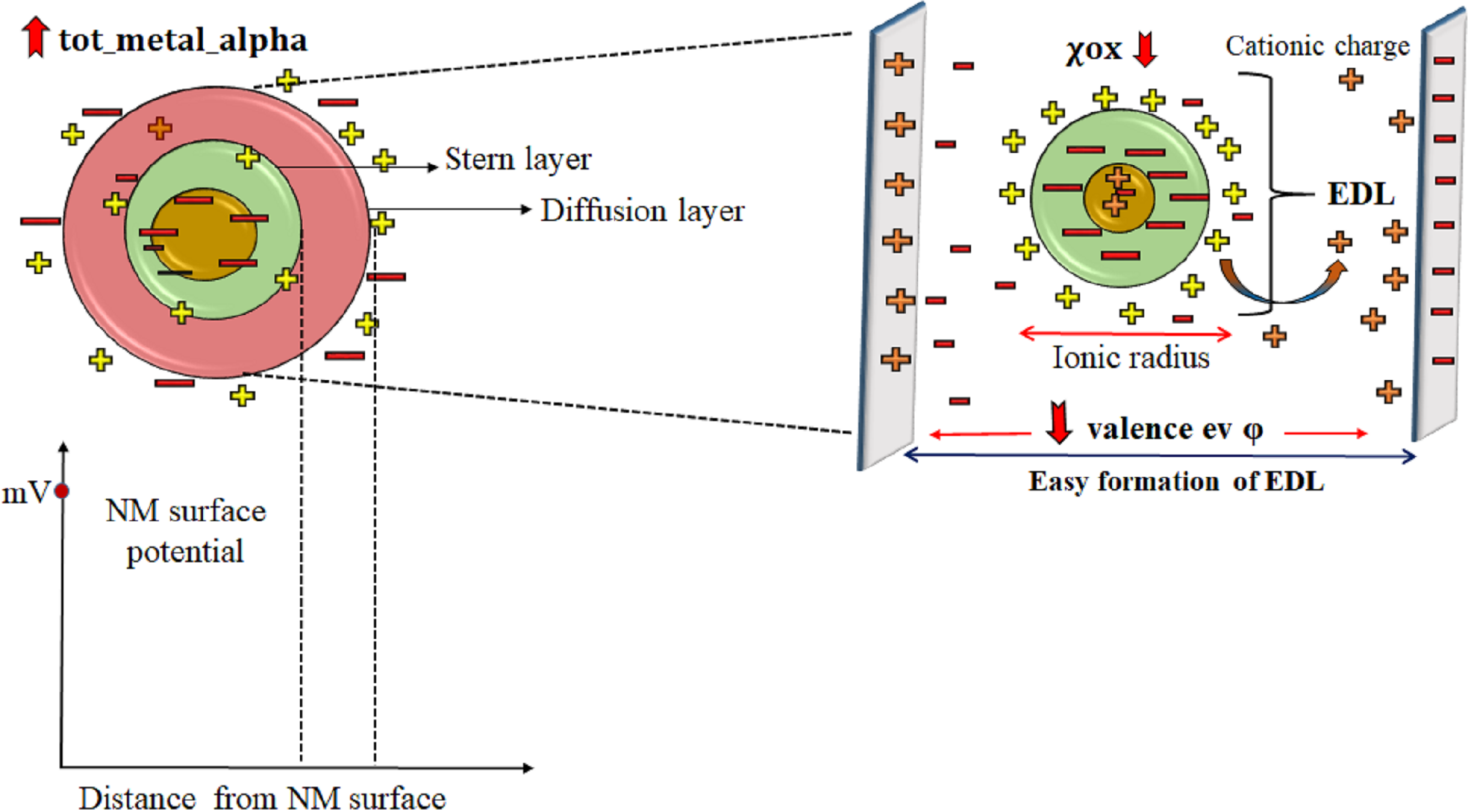
Zeta potential formation and influence phenomenon in respect to the modeled descriptors.

The zeta potential is the key parameter from the regulatory point and can directly affect the NPs’ behavior in solution and their interaction with biological organisms (Figure 4). 18 MeOx NPs were modeled against the zeta potential endpoint to obtain the partial least squares (PLS) model with one latent variable (LV).

#### Model 1 (PLS)


[1] $$\begin{array}{c} \zeta =35.9157-8.4317\chi \text{ox}+2.0002\text{tot\_metal\_alpha}\\ -0.1854\text{ valence electron potential}\\ N_{\text{train}}\;=\;13;\;R^{2}\;=0.80;\;Q_{\text{(LOO})}^{2}\;=\;0.67;\;N_{\text{test}}\;=\;5;\;Q_{f1}^{2}\;=\;0.68;\\ Q_{f2}^{2}=0.67;\text{ LV}=1;\;F=44.20;\;p=0.002 \end{array}$$

Model 1 considers three descriptors to evaluate the influence of the zeta potential based on basic attributes. Here, *N*_train_ and *N*_test_ stand for the number of training and test set compounds, respectively. *R*^2^ is the determination coefficient; 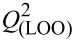 is the leave-one-out cross validation determination coefficient. Again, 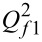 and 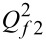 were calculated for external data predictions. The model parameters suggest the good predictive ability of the developed model as it passes various statistical criteria [34]. The descriptors depicted in the model also interpret the influence of the zeta potential as discussed below.

The descriptor “χox” pertains to the oxidation number of the metal, which represents the hypothetical charges within an atom. The zeta potential decreases as the oxidation number increases, as indicated by the negative coefficient of the descriptor. A lower (negative) oxidation number indicates a higher electronegativity of the metal, which determines the electron distribution in a molecule. The metal’s electronegativity also influences the catalytic property of the cationic form and the surface charge formed around the metal oxide surface. The highly electronegative surface of MeOx NPs [39] affects their behavior and stability, thus determining the net charge of ions in a given medium. Certain MeOx NPs are unstable and tend to agglomerate. NPs attract negative or positive ions from the medium to build a diffusion double layer. The electronegativity of the NPs also depends on the pH value of the medium [40]. In colloidal solutions, negatively charged metal oxides decrease the zeta potential, which reflects stability based on the aggregation phenomenon. This is well observed in MeOx NPs, where an increase in the oxidation number (χox) decreases the zeta potential. In WO_3_ NPs, the χox value is 6 and the zeta potential value is −23 mV; for NiO NPs, the χox value is 2, and the zeta potential value is 34.4 mV.

The “valence electron potential” (−*eV*) determines the elements’ reactivity and is based on the charge of the valence electrons and the ionic radius:




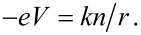




Here, *k* is a proportionality factor expressing the energy of the valence electrons in electronvolts. *n* is the valence, and *r* is the ionic radius.

This descriptor negatively contributes to the zeta potential suggesting that with the increase of the valence electron potential of the metal, there will be a decrease in zeta potential value. This has been observed in Mn_2_O_3_ NPs, which have a valence electron potential value of 220*eV* and a zeta potential of −15.9 mV. Co_3_O_3_ NPs show the opposite result; the decrease in the valence electron potential value (38*eV*) shows an increase in zeta potential value (22.6 mV). MeOx NPs with large ionic radius tend to have low valence electron potential, as it is inversely proportional to the ionic radius of the NPs. NPs with lower valence potential allow for an easier formation of the electrostatic double layer (EDL). If the solution with NPs shifts to lower ionic strength, then the zeta potential increases as the EDL expands to balance the electrostatic force, thus allowing for the dispersion of NPs.

The descriptor “tot_metal_alpha” defines the core environment of the metal. It also defines the molecular bulk of the metal oxide. This descriptor has vital characteristics that are heavily influenced by the number of metals present in the metal oxide. Furthermore, the electronegativity of the metal is a crucial factor in determining the surface charge and stability of the NPs in the solution. The positive regression coefficient suggests that an increase in the surface charge of the metal helps the NPs to remain dispersed in the media and thus avoids flocculation. This phenomenon is observable in Yb_2_O_3_ NPs with a high tot_metal_alpha value (13.6) and the highest zeta potential (46 mV); in contrast, SnO_2_ NPs with a descriptor value of 2.88 have a zeta potential value of −20.5 mV.

### QSPR model for cell damage

#### Model 2 (PLS)


[2] $$\begin{array}{l} \text{cell damage}=-1.681-1.11\frac{\Sigma \chi }{n\text{O}}+0.295\frac{{\text{sq}}_{{\text{sum}}_{\text{epsilon}}}}{N}\\ +0.318\;D1_{\text{metal}}-0.263\text{ Metal alpha}\\ +0.035\text{ time of exposure}+0.00057\text{ zeta potential}\\ \text{+0.0057 }{\text{Electrons}}_{\text{ActiveM}}-0.079\text{ valence}\\ N_{\text{train}}=110;\;R^{2}=0.62;\;Q_{\text{(LOO})}^{2}=0.54;\;\bar{rm_{\text{LOO}}^{2}}=0.389;\\ \Delta rm_{\text{LOO}}^{2}=0.246;\;N_{\text{test}}=21;Q_{f1}^{2}=0.653;\;Q_{f2}^{2}=0.652;\\ \bar{rm_{\text{LOO}}^{2}}=0.532;\Delta rm_{\text{LOO}}^{2}=0.183;\text{ LV}=7;\\ {\text{MAE}}_{\text{test}}=0.206;\;F=19.87 \end{array}$$

Model 2 utilizes eight descriptors to evaluate crucial attributes that can impact cell damage. Equation 2 shows the number of compounds used in the training and test sets represented by *N*_train_ and *N*_test_, respectively. Additionally, *R*^2^ and 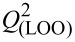, the determination coefficient and leave-one-out (LOO) cross-validation coefficient, were employed. Furthermore, external data prediction calculations were made using 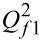 and 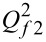. The model parameters demonstrate exceptional predictive ability, meeting various statistical criteria [34]. The descriptors used in the model were well interpreted and are comprehensively discussed in a later section. Note that the zeta potential has appeared as a significant descriptor in defining the cell damage. On removal of zeta potential as a descriptor, the model quality decreases. Predictions from one model as a descriptor for another model are made to fill the data gap or to determine the missing values. This approach is similar to the imputation methodology, which creates a model embedded within another model. Instead of using dummy variables for quantitative prediction, a useful imputation method can predict various types of inputs. It is worth noting that many existing works utilize imputation techniques [41]. In QSAR studies, it is not unusual to use a model-derived prediction as a descriptor for the development of other models or for prediction when the endpoint has been tested under different experimental or varying conditions (as in the case of interspecies modeling). This approach is reliable and aims to establish a correlation between different conditions to fill the data gap.

### Chemical read-across analysis

The developed QSPR (PLS) model for dataset 2 provided eight descriptors that were utilized for read-across predictions. Three similarity-based prediction methods, namely Euclidean distance (ED)-based, Gaussian kernel (GK) similarity-based, and Laplacian kernel (LK) were employed. Upon optimizing the dataset, it was concluded that the read-across based on the Euclidean distance (RA-ED) function outperformed the others, as shown in Table 1. The Read-Across v4.0 software [42] was utilized for this work. After performing RA, the resultant 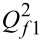 increased from 0.65 to 0.766.

**Table 1:** Results for read across prediction using different similarity-based approaches.

Feature combinations	Hypothesis	Hyper parameters				Statistical parameters			
	
		σ	γ	Distance threshold	Similarity threshold	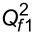	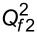	MAE	RMSEP

Model 2 (132 MeOx NPs) 8 descriptors (7 LVs)	RA-ED	1.75	1.75	1	0	0.766	0.765	0.177	0.252
	RA-GK					0.764	0.763	0.178	0.254
	RA-LK					0.724	0.724	0.195	0.274

### Interpretation of the descriptors

The periodic table descriptor ∑χ/*n*O stands for the total metal electronegativity in a specific metal oxide relative to the number of oxygen atoms. This descriptor takes into account the crucial role of oxygen atoms in causing cell damage. With regard to the cell damage endpoint, this descriptor has a negative effect, indicating that an increase in the number of oxygen atoms compared to the electronegativity sum results in a lower ratio of the descriptor. Thus, a high concentration of oxygen atoms in the metal oxide can expedite the oxidative damage process, leading to the production of more reactive oxygen species (ROS) and causing more cell damage. CoO NPs show that a high ∑χ/*n*O value (1.88) leads to less cell damage (−4.38), whereas a low value (∑χ/*n*O = 0.77) leads to more cell damage (−2.50) as observed for TiO_2_ NPs. The production of ROS can enhance the catalytic activity of Fenton/Fenton-like reactions, but can also result in cellular damage [16]. ROS can break down the basic components of the cell, including DNA, proteins, and lipids. ROS can cause double-strand breaks in DNA by converting guanine to 8-oxoguanine. This conversion can lead to mispairing with adenine, resulting in transversion mutations. Proteins can also be damaged when their amino acid side chains are oxidized by ROS. Exposure of lipids to ROS can result in lipid peroxidation, which can cause cell damage and generate reactive by-products that further damage the cell.

The second-generation periodic table-based descriptor “sq_sum_epsilon/*N*” (∑ε/*N*)^2^ stands for the sum of electronegativity of the atoms of the metal oxide, which is calculated based on the electronegativity count (∑ε) of the oxides, scaled by the number of atoms:




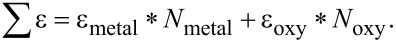




Here, ε_metal_ and ε_oxy_ are the electronegativity count of metal and oxygen atoms, respectively, and *N*_metal_ and *N*_oxy_ are, respectively, the number of metal and oxygen atoms. The positive coefficient of the descriptor in Equation 2 indicates that an increase in electronegativity favors the rise in cell damage as in CuO nanoparticles, where a high (∑ε/*N*)^2^ value (9.93) causes more cell damage (−2.87), whereas Sb_2_O_3_ nanoparticles with low electronegativity ((∑ε/*N*)^2^ = 0.018) are less toxic (−4.625). Because of the high electronegativity, the atoms pull electrons from their neighboring atoms or molecules, leading to the development of an electrostatic bond with proteins in biological systems. The high electronegativity also influences the formation of metal cations. The increase of catalytic properties of metal cations enhances the toxicity through the generation of ROS, causing damage to cell membranes [16]. The high electronegativity helps in removing electrons from molecules, producing free radicals. Free radicals are unstable and highly reactive. These short-lived radicals are unable to leave the sub-cellular location where they are generated without being reduced, leading to oxidative damage [43]. The presence of high-electronegativity metals in the cellular membrane can lead to the leakage of cellular content [22].

The “D1_metal_” descriptor signifies the total number of metal atoms in the MeOx NP composition. An increase in the number of metals can have a detrimental effect on cells by impacting ROS generation. The positive coefficient of the D1_metal_ descriptor indicates that an increase in the metal fraction in MeOx NPs causes more cell damage (−2.63) as observed in Fe_3_O_4_ NPs (D1_metal_ = 3). In contrast, CoO NPs with a low metal fraction (D1_metal_ = 1) nanoparticles cause less cell damage (−4.375). Metal ions can generate reactive hydroxyl radicals, resulting in oxidative damage to proteins. Moreover, they can bind non-specifically to amino acid residues and replace existing metal ions at active sites of enzymes, leading to abnormal protein folding. Protein aggregation diseases are a type of neurodegenerative diseases that occur when proteins lose their structure and are deposited in the brain. These diseases are the most common type of neurodegenerative diseases. Many of these structures are highly toxic to cells [44]. The folding of proteins also causes damage to the immune system, because certain structures do not induce the production of antibodies [45].

The descriptor “Metal alpha” (α_metal_) defines the core environment of the metal. This descriptor represents the ratio of the number of core electrons to the number of valence electrons. The Metal alpha descriptor describes the electron density of the metal. This descriptor is calculated using Equation 3:


[3] $$\alpha_{\text{metal}}=\lambda *\mu .$$

Here, λ is (*Z*_metal_ − *Z*_vmetal_)/*Z*_vmetal_ and μ is 1/(PN_metal_), where *Z*_metal_ is the atomic number, *Z*_vmetal_ stands for the valence electrons of the metal, and PN_metal_ stands for the periodic number in the periodic table. The negative coefficient of the descriptor signifies the low electron affinity of the metal oxide to accept electrons. This means that the metal has a propensity of having a cationic charge, which leads to the catalytic power of metal cations. For example, in WO_3_, the metal alpha value is 7.2 and cell damage is −4.57. In contrast, Al_2_O_3_ with a metal alpha value of 1.66 causes higher damage to cells (−2.8). Metal cations are more harmful than normal nanoparticles. This is because their electropositivity and inherent toxicity increase significantly with atomic weight. In addition, the formation of metal–ligand bonds has a direct impact on the metal’s toxicity. Furthermore, it is a well-established fact that each metal has an affinity constant for various ligands, which means that most metal cations can form stable complexes with a wide variety of ligands, further increasing their potential toxicity.

In the field of physical chemistry, the zeta potential is a crucial parameter that measures the surface charge of particles relative to their size. In colloidal systems, the zeta potential is widely used as an indicator to reflect the stability. It is important to note that NPs with higher positive charges can be more harmful than those with higher negative charges. Moreover, positively charged NPs interact more significantly with cells, leading to greater cell damage. Another crucial factor to consider is that NPs with a higher zeta potential, regardless of their charge, are more easily absorbed by cells due to the electrostatic interaction between dispersed particles and the effective electric charge on the surface of the NPs [40]. This feature is particularly relevant to their biological activity, especially their ability to bind to and be absorbed by cell membranes. For instance, Cr_2_O_3_ NPs have a high zeta potential (2130 mV) and a high cell damage propensity, whereas Y_2_O_3_ NPs with a low zeta potential (−23 mV) cause less damage to cells (−4.5). The increase in zeta potential enhances the accumulation of nanoparticles on the surface of cells. The intensity of accumulation determines the toxicity of the nanoparticles. The concept of zeta potential plays a vital role in adhesion to the hydro–water interface and solid surfaces, providing an idea about the viability and permeability of the cell membrane under stress. As most of the cell surface carries a negative charge, metals with higher zeta potential can easily enter the cell and increase the production of ROS. Also, they can have a mechanical effect on the membrane, leading to depolarization of the membrane and cell damage.

The “Electron Active M” descriptor is a representation of the number of electrons that an active metal possesses. Active metals are known for their quick and robust reactions owing to the electron arrangement in their structure. These metals contain free electrons in their outermost shell that can readily create a cation by interacting with other atoms and initiating a chemical reaction. The delocalized electrons can easily interact with macroproteins, leading to the acceleration of damage to the biological membrane. A positive coefficient of Electron Active M indicates more oxidative stress and more damage to the cell due to an increase in free radicals. WO_3_ has a high descriptor value of 74 resulting in high cell damage (−2.8), while Cr_2_O_3_ NP has a low descriptor value of 24 leading to low cellular damage (−4). Transition metals are capable of forming coordinate complexes with the imidazolyl group of histidine. These metal ions are redox-active and can play a crucial role in the production of ROS within the cell. The reduced forms of these redox-active metal ions are involved in the Fenton reaction, which generates hydroxyl radicals from hydrogen peroxide. Similarly, the Haber–Weiss reaction involves the oxidized forms of redox-active metal ions and superoxide anions, which generate the reduced form of the metal ion. This reduced form can then be coupled to Fenton chemistry to produce hydroxyl radicals. ROS further accelerate the damage of the cell.

“Valence” (*V*) is a factor that contributes to cell damage. It indicates the number of electrons in the outermost shell of an atom that are available for chemical bonding and is similar to other descriptors that provide information about free electrons. The insights obtained from the developed model 2 strongly suggest that an increase in valence (7) leads to a decrease in cell damage (−3), as observed in MnO_3_ NPs. This is supported by the negative regression coefficient of the descriptor. Conversely, a low valence (2) leads to greater cell damage (−2), as seen in ZnO NPs. Atoms with fewer electrons in their outer shell tend to lose them and become metal cations, which can damage cells [16]. Cations aid in the transportation of metal ions across the cell surface by interacting with its negatively charged surface. Unfortunately, this interaction can lead to DNA damage through processes such as delocalization, redox chemistry, and the generation of ROS.

Our research aimed to examine how the time of exposure to metal oxide affects cell damage, regardless of other physiochemical properties of MeOx NPs. Our findings indicate that exposure time plays a crucial role in cell damage. Prolonged exposure times increase the damaging potential. For instance, exposing cells to WO_3_ NP for 7 h resulted in a cell damage score of −2.75. In contrast, exposure to Yb_2_O_3_ for only 1 h resulted in a score of −3.5. These results demonstrate the significance of considering exposure time when evaluating the potential risks of metal oxide exposure. When living organisms are exposed to NPs for an extended period of time, inflammatory conditions can occur that lead to physical, muscular, and neurological degeneration, or increased intensity of oxidative stress. This happens because longer exposure times enhance the toxicity mechanism of NPs. In contrast, short-term exposure does not affect significantly the cells. NPs can induce oxidative stress by impairing antioxidant defenses in humans when they are chronically exposed to NPs.

### Importance of the zeta potential as a descriptor

The developed QSPR model without zeta potential descriptor shows *R*^2^ = 0.47 and 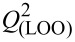 = 0.34, which is well below the desired acceptance criteria. The obtained results indicate that the fitting and robustness of the developed model without the presence of the zeta potential descriptor is unsatisfactory. Therefore, to achieve the fit and predictive power of the model, we included the zeta potential descriptor along with the other seven descriptors. In the presence of zeta potential, the statistical quality and internal validation metrics increased (*R*^2^ = 0.62 and 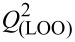 = 0.54) showing the stability and predictive ability of the model.

### Utilization of the metal oxide cell damage knowledge for cancer treatment

**Figure 5:**
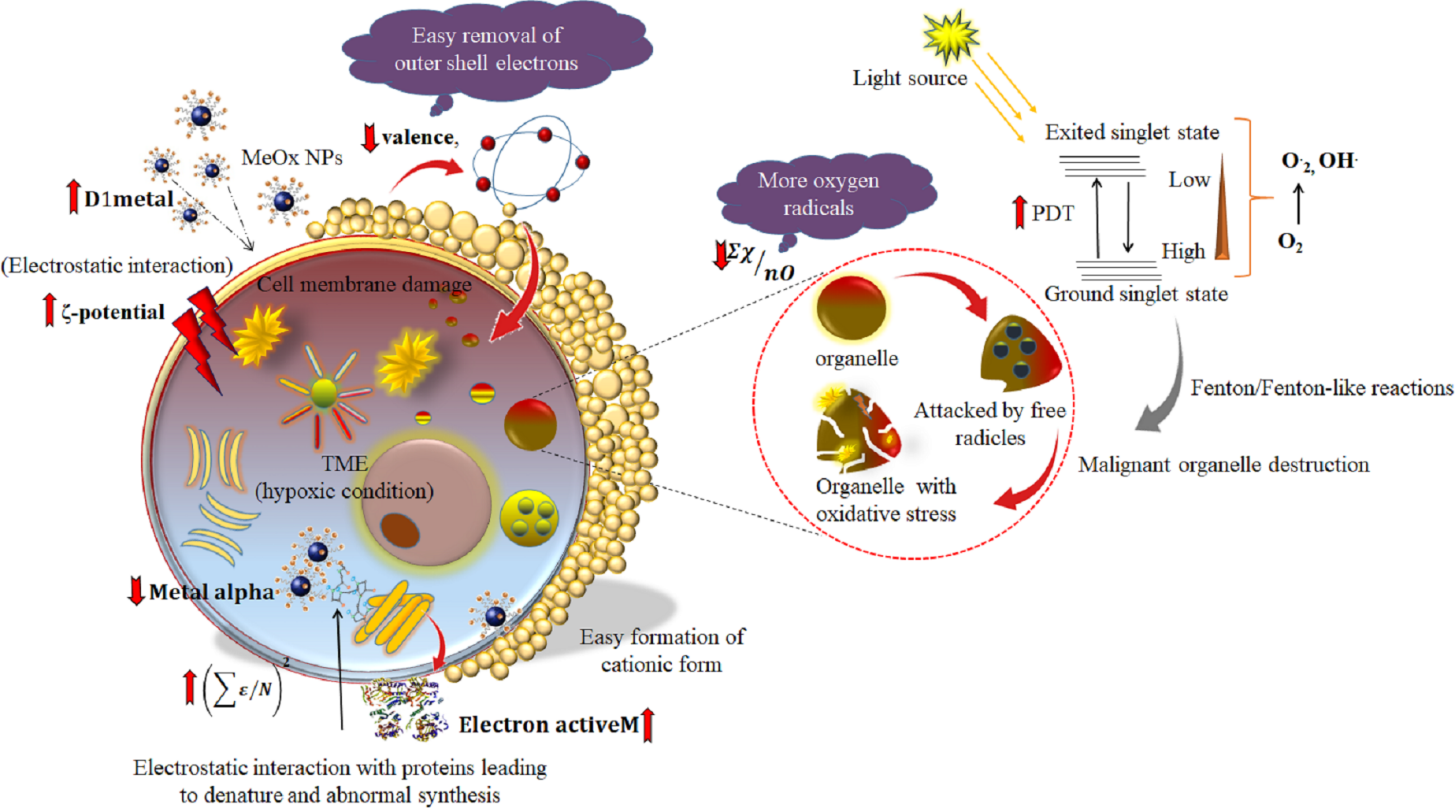
Interpretation of descriptors with respect to cell damage (endpoint) in cancer cells.

NPs have shown immense potential in treating various diseases owing to their small size and high surface-to-volume ratio, which makes them effective drug delivery systems. Metal NPs can lead to greater signal amplification, greater sensitivity, and higher detection. However, NPs with properties that generate ROS can increase cell damage. In cancer cells, rapid proliferation leads to an imbalance of oxygen, abnormal structure, and blood supply, making the tumor microenvironment (TME) prone to hypoxic conditions [46]. Insufficient oxygen reduces ROS generation, which decreases the efficacy of oxygen-dependent therapies, such as photodynamic therapy (PDT), chemodynamic therapy (CDT), and radiation therapy. The information derived from the positive contribution of the D1metal descriptor (model 2) draws attention to the fact that metal oxides are good candidates for generating oxidative stress in cells. The ∑χ/*n*O descriptor suggested a higher oxygen requirement for damaging the cells. It indicates that a higher fraction of oxygen in the metal oxide nanoparticles can increase the sensitivity to PDT. Furthermore, transition metals can catalyze Fenton/Fenton-like reactions [47], generating highly oxidative species that can kill tumor cells. The electronegativity of the metal oxides helps the NPs in crossing the cell membrane. The formation of metal cations can also affect the pH value of the cell and increase the catalytic properties of metal oxides, thereby increasing ROS generation. Tumor cells have a mechanism for dealing with hypoxia, acidosis, and high glutathione (GSH) levels, which promote drug resistance, especially for ROS-dependent drugs (Figure 5). However, metal oxides can change the TME conditions by supplying oxygen and suppressing hypoxia-inducible factor 1 and CD39/CD73 in T cells, which reduces the immunosuppression effect of tumors.

### Comparison with previously published literature

This study successfully develops a QSPR model with a cell damage endpoint that uses the zeta potential value as a descriptor. The descriptor was calculated using another model that used the zeta potential as the endpoint (Y-response) for its QSPR model development. The QSPR models were developed with simple periodic table-based descriptors that do not depend on size or any other experimental conditions. These descriptors are easy to calculate, less expensive, and can be calculated by anyone without the need for expert personnel.

The study provides in-depth knowledge about the properties and causes of toxicity of nanoparticles using simple regression-based models. It is important to note that a direct comparison with a previous study by Toropova [27] is not possible because of the different data division methods (five random splits), the use of different types of descriptors (optimal nano-descriptors), and the dissimilar modeling methods (Monte Carlo method). However, it is clear that the statistical metric values for the developed model in the present study are similar to those of the previous study (the best-split results only shown) as presented in Table 2. Furthermore, we have proposed an effective mechanism to treat cancerous cells with the cell-damaging properties of MeOx NPs.

**Table 2:** Comparison of the statistical parameters with a previous study.

Sl. no.	*Q* ^2^	*R* ^2^_train_	s_train_	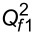	*R* ^2^_test_	MAE_test_	*S*_test_

current study	0.538	0.621	0.361	0.768	0.767	0.181	0.377
previous study (best split) [27]	0.486	0.512	0.387	—	0.822	—	0.318

## Conclusion

The impact of nanoparticles on cell membranes has been thoroughly examined through nanotoxicological research and in vitro modeling [48–49]. While the toxicity endpoint is a well-explored topic, it is crucial to investigate non-fatal endpoints such as cell damage. The zeta potential is a widely used parameter to characterize the properties of nanoparticles. However, cell membrane damage is influenced by various factors, including exposure time and dose. Thus, this study aimed to establish a relationship between the properties of nanoparticles and their toxicity, with a focus on cell membrane damage.

The study was divided into two parts. The first part involved modeling nanoparticles against the zeta potential to determine the features that can alter their properties. The second part focused on the elements that can influence toxicity and damage to the cell membrane. Both the QSPR model for the zeta potential and another model against cell damage were developed using periodic table-based descriptors. The QSPR model (zeta potential) was able to predict the zeta potential for MeOx NPs without experimental values. The developed models showed good predictivity and robustness, confirming their effectiveness.

The features obtained from the models suggest that surface charge and electronegativity play a role in altering the zeta potential. Additionally, an increase in oxygen count, electronegativity, formation of cationic charge, and an increase in zeta potential can influence cell membrane damage. Based on these findings, the authors propose that the damaging power of metal oxide nanoparticles can be harnessed in treating cancerous cells. This study not only identifies the features required to enhance the properties of nanoparticles but also provides knowledge for treating cancerous cells through cell damage techniques. The study can pave the way for researchers to use nanoparticles in clinical practice with confidence.

## Supporting Information

Supporting Information File 2: The sheet details information on the metal’s oxides with zeta potential and cell damage endpoint along with the external set used in the present work. Supporting Information File 1: PLS graphs for both cell damage and zeta potential data.

File 1 Additional experimental data.

File 2 Additional figures.

## Data Availability

All data that supports the findings of this study is available in the published article and/or the supporting information to this article.
