# Supplementary material for: Exploring the relationships between physiochemical properties of nanoparticles and cell damage to combat cancer growth using simple periodic table-based descriptors
Source: Beilstein J Nanotechnol. 2024 Mar 12;15:297–309. doi: 10.3762/bjnano.15.27 (PMC10949013; doi:10.3762/bjnano.15.27)
Supplement: File 2 — Additional figures. [file Beilstein_J_Nanotechnol-15-297-s002.pdf]

BEILSTEIN JOURNAL OF NANOTECHNOLOGY

Supporting Information

for

Exploring the relationships between physiochemical properties of nanoparticles and cell damage to combat cancer growth using simple periodic table-based descriptors

Joyita Roy and Kunal Roy

Beilstein J. Nanotechnol. **2024,** *15,* 297–309. doi:10.3762/bjnano.15.27

Additional figures

License and Terms: This is a supporting information file under the terms of the Creative Commons Attribution License (https://creativecommons.org/licenses/by/4.0). Please note that the reuse, redistribution and reproduction in particular requires that the author(s) and source are credited and that individual graphics may be subject to special legal provisions.
The license is subject to the *Beilstein Journal of Nanotechnology* terms and conditions: (https://www.beilstein-journals.org/bjnano/terms)

SUPPLEMENTARY SECTION (S1)

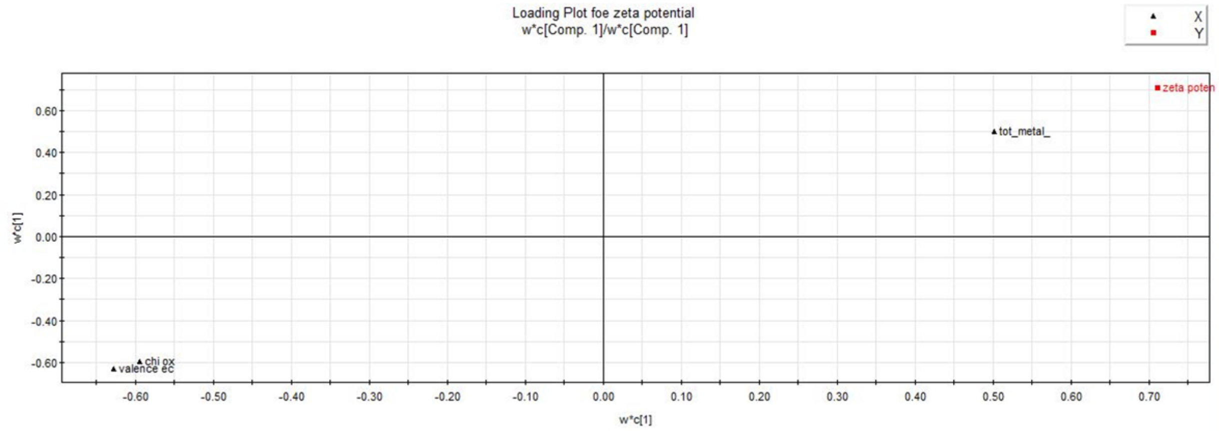

A

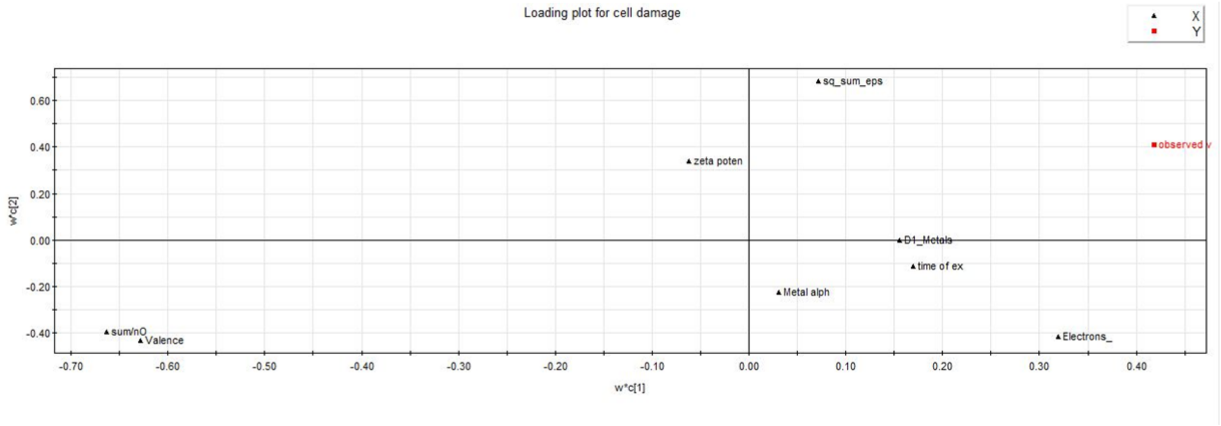

B

Figure S1: Loading plot of zeta potential data (A) and cell damage dataset (B).

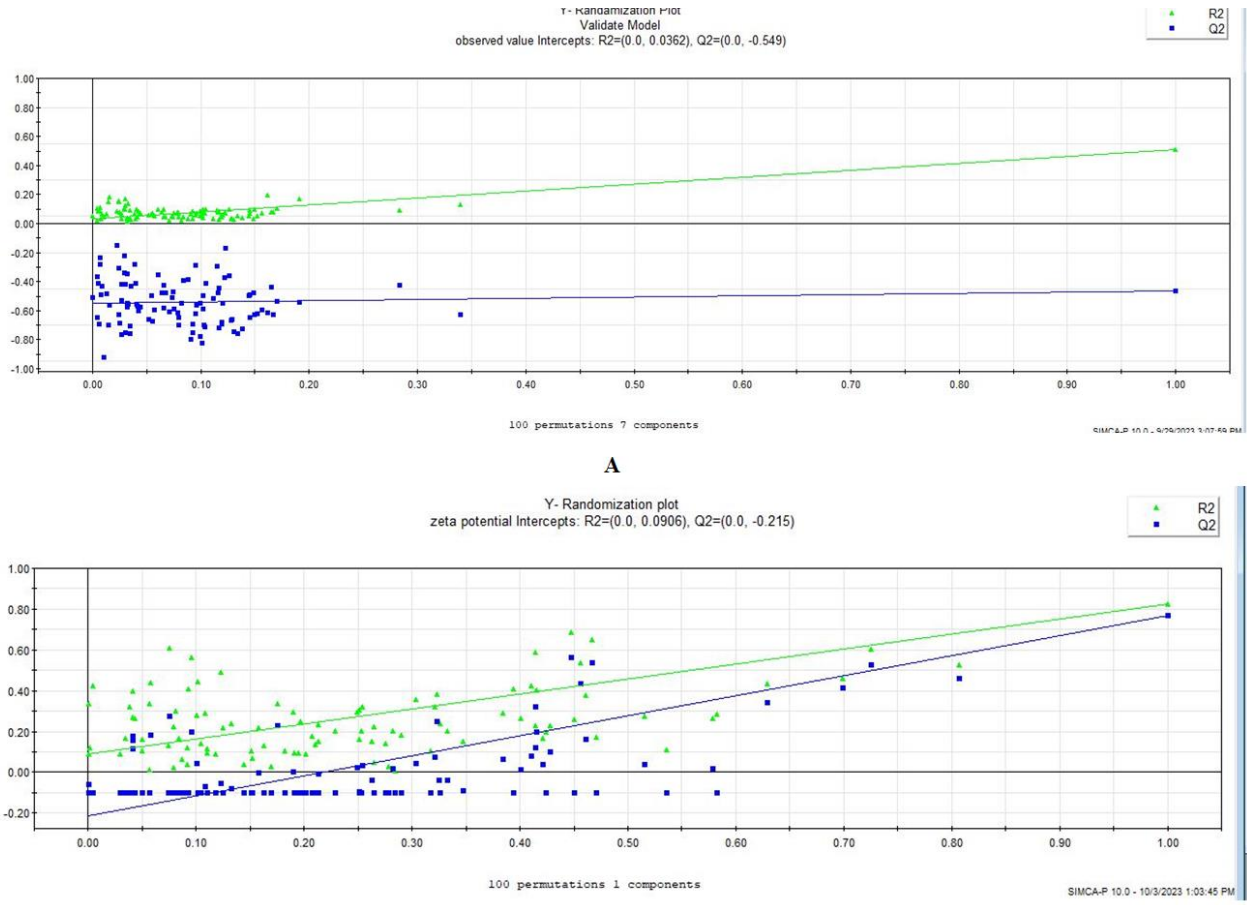

A

B

Figure S2: Y-randomization plot of zeta potential data (A) and cell damage dataset (B).
